# Assessment of Pesticide Residues in Flesh of *Catla catla* from Ravi River, Pakistan

**DOI:** 10.1155/2014/708532

**Published:** 2014-06-05

**Authors:** Mobeen Akhtar, Shahid Mahboob, Salma Sultana, Tayyaba Sultana, Khalid Abdullah Alghanim, Zubair Ahmed

**Affiliations:** ^1^Department of Zoology, GC University, Faisalabad, Pakistan; ^2^Department of Zoology, College of Science, King Saud University, P.O. Box 2455, Riyadh 11451, Saudi Arabia

## Abstract

The levels of dichlorodiphenyltrichloroethane (DDT), dichlorodiphenyldichloroethylene (DDE), endosulfan, endosulfan sulfate, carbofuran, and cartap which were estimated in the flesh of *Catla catla* sampled from ten sites of Ravi River between its stretches from Shahdara to Head Balloki were studied to know the level of contamination of the selected pesticides by GC-ECD method. All fish samples were found contaminated with different concentrations of DDT, DDE, endosulfan, and carbofuran; however, DDT and DDE concentrations were more than the maximum residue limits (MRLs) about food standards, while endosulfan sulfate and cartap were not detected. Pesticide concentrations in the fish flesh were ranged from 3.240 to 3.389 for DDT, 2.290 to 2.460 for DDE, 0.112 to 0.136 for endosulfan, and 0.260 to 0.370 **μ**g g^−1^ for carbofuran. The findings revealed that the pesticide concentrations in the fish flesh decreased in the order: DDT > DDE > carbofuran > endosulfan. After Degh fall and After Hudiara nulla fall river sampling sites were found severely contaminated. It is proposed that a constant monitoring programs are needed to be initiated to overcome the present alarming situation.

## 1. Introduction

Persistent organic pollutants (POPs) are a major group of hazardous chemicals having anthropogenic origin and three main characteristics: persistence, bioaccumulation, and long range transport [1]. In the recent years, there has been a growing interest in these chemicals due to their potential toxicity and adverse impacts on human health [2]. The Stockholm Convention on POPs (2001) is an outcome of this growing concern about the persistent organic pollutants. As per the Stockholm Convention, POPs include nine organochlorine pesticides (OCPs) and three industrial chemicals or by-products. The nine pesticides targeted by the Stockholm Convention were produced internationally and used on agricultural crops or for public health vector control. By 1970s, these pesticides were either banned or restricted in many countries. Though banned, these chemicals are still being used in some of the developing countries owing to their low cost and versatility in industry, agriculture, and public health [3]. The use of banned pesticides is still not only continued in Pakistan but also continued all over the world as a number of recent works support the presence of pesticides in biota especially in 24 aquatic life, such as India [4] and Pakistan [5]. There are benefits of using pesticides, but they also have drawbacks of potential toxicity to many other nontarget species, including man, fish, and other aquatic fauna [6]. From the areas of applications, these pesticides find their way to nearby aquatic bodies through leaching and cause contamination of surface as well as ground water [5]. In aquatic ecosystems, compounds such as pesticides with low water solubility and high liposoluble are forced to seek organic lipid containing material [7]. With long-term use of the pesticides, they can accumulate in the environment [8], which can result in differential effects based on the concentrations. Appropriate use of pesticides based on the recommendation is generally expected to cause little adverse impact on the environment [9]. Consequently, community concerns regarding the potential effects of pesticides on nontarget organisms have increased immensely [10]. Majority of organochlorines are banned in Pakistan but still they are in use and are causing residual and other toxic effects to fauna and flora [11, 12].

Ravi River is the smallest among the five main eastern tributaries of the Indus, which takes its origin from India and in Pakistan it enters near village Tadyal, Kot Naina, Tehsil Shakargar of Sialkot. After flowing about 560 km, it joins River Chenab near village Sayyal Faqir, Sidhnai, Tehsil Kabirwala. In addition to its surface run-off upstream water, it also receives water from the Qadir Abad link canal and upper Chenab canal between its stretches from Shahdara to Balloki Headworks. Raw domestic sewage and untreated industrial effluents originating from the cities of Lahore, Sheikhupura, and Kalashahkaku are dumped into the Ravi River by main tributaries of Hudiara drain and Degh fall. Hudiara drain originates from Batala in Gurdaspur district, India, and enters into Pakistan at village Laloo which is approximately 44 km away from Lahore city.

Keeping in view the environmental significance of organochlorine and carbamates in Pakistan context and highly polluted status of the Ravi River, the study was undertaken to assess the residual presence of selected pesticides the flesh of* Catla catla* in Ravi river which is one of the indigenous fish in the Indo-Pak regions. The results obtained may be made use of as a baseline data in developing effective remedial measures to improve the water quality status of the river.

## 2. Materials and Methods

### 2.1. Study Area and Sample Collection

The Ravi River along the India-Pakistan border meanders substantially in the alluvial plains of the Amritsar and Gurdaspur districts of Punjab before entering Lahore, Pakistan. The pollution levels in the river discharge are reportedly very high, which is attributed to careless disposal of large amounts of industrial and agricultural wastewater and faulty drainage system in both countries. A 72 km stretch of the Ravi River from Lahore Siphon to Bulloki Headworks indicates heavy contamination of the water and sediment with different pesticides. The river sediments are highly contaminated and have become a secondary source of pollution of the river water, even though some controls over unauthorized discharges into the river have been checked. The effect of pollution was evaluated on fish samples of* Catla catla *from predetermined locations nearby Head Balloki in triplicates.

**Figure 1:**
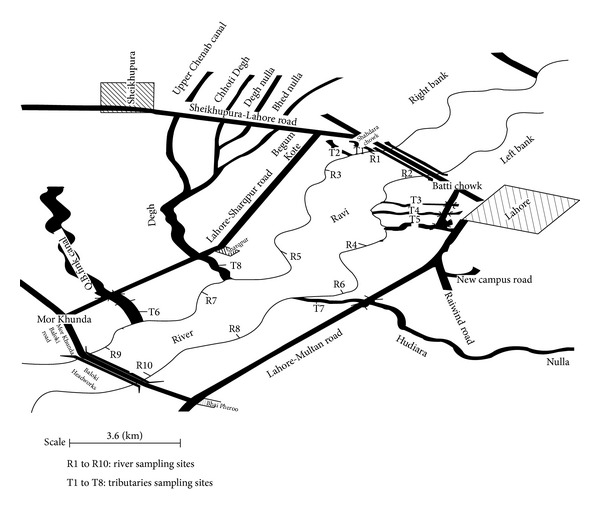
Map of area under study showing sampling sites.

To find out the organochlorine (dichlorodiphenyltrichloroethane, dichlorodiphenyldichloroethylene, endosulfan, and endosulfan sulfate) and nitrogen containing pesticide residues (carbofuran and cartap) in* Catla catla*, fish samples were taken for one year on a fortnightly basis from ten sampling sites along right and left banks of Ravi River between its stretches from Shahdara to Head Balloki, namely, River site (R1): Shahdara bridge downstream, right bank (R B), R2: Shahdara bridge downstream, left bank (L B), R3: After Farrukhabad nulla fall (R B), R4: After Bakar mandi nulla (L B), R5: Before Degh fall (R B), R6: Before Hudiara drain (L B), R7: After Degh fall (R B), R8: After Hudiara drain (L B), R9: Balloki Headworks upstream (R B), and R10: Balloki Headworks upstream (L B), respectively. Map of the study area with sampling sites is presented in Figure 1. Fish samples were brought to the Fisheries Research laboratory, Department of Zoology, GC University, Faisalabad. Three fish samples were collected and processed for the detection of selected pesticide residues. Fish samples were washed with dechlorinated water, descaled, and dissected. Muscle tissues from fish samples were taken to cut into small pieces and frozen at −20°C until analysis [13].

The extraction and cleanup of dichlorodiphenyltrichloroethane (DDT), dichlorodiphenyldichloroethylene (DDE), endosulfan, endosulfan sulfate, carbofuran, and cartap in fish samples were done with some modifications in the method [14].

Calibration curves of all standard pesticides were prepared with the help of computer software Turbochrome (Perkin Elmer, Inc., USA) and limit of detection was calculated by computer software Super cal-5. LOD for the OPs—was 0.1 ng/g lipid, and the limit of quantification (LOQ) was 0.3 ng/g. Quality-assurance measures applied in the laboratory included rigorous contamination-control procedures (strict washing and cleaning procedures), monitoring of blank levels of solvents, equipment and other materials, analysis of procedural blanks, recovery of spiked standards, monitoring of detector response and linearity, and analysis of a reference material.

*1 L (extract 1).* 50 mL of acetonitrile was again added to the mixture and shaken for 2 minutes, composite was taken, and 10 g of anhydrous sodium sulpate (Merck) was added to disintegrate it. A mixture of fish flesh and anhydrous sodium sulphate was blended and mixed with a spatula until both were well mixed. A 20 mL of hexane (Merck) was added to the mixture, followed by the addition of 100 mL of acetonitrile (Merck, isocratic grade) saturated with hexane. The mixture was shaken for 2 minutes. Acetonitrile was decanted into a separatory.

For further two times after adding 50 mL of acetonitrile followed by shaking for 2 minutes in both cases, acetonitrile was then drained into separatory funnels (extracts 3 and 4). All extracts were combined and 500 mL of distilled water was added to wash them. A 40 mL of saturated sodium chloride (Merck) solution and 50 mL of hexane were added to the extract. Separatory funnel was kept in a stand until two distinct layers were formed. The aqueous layer was drained into another 1L separatory funnel. A 50 mL of hexane was added to this aqueous layer (hexane extract 1). The mixture was again extracted with 50 mL of hexane (hexane extract 2). Combined hexane extracts (hexane extracts 1 and 2) were passed through a plug of 10 g of anhydrous sodium sulphate. The solvent (hexane) was evaporated using a rotary evaporator (Buchi-R-200) to get the residue. A 10 mL of methanol (Merck) was added to the residue and diluted by adding 25 mL of distilled water.

#### 2.1.1. Cleanup

Solution of sample extract was transferred on to preconditioned C-18 solid phase extraction bond column. Pesticide residues were recovered by eluting the column with 5 mL portions of hexane for three times. Elute was concentrated to 2 mL using rotary evaporator and the concentrated elute was now ready for injection into a gas chromatograph (Model Number 7900, Hitachi, Japan).

#### 2.1.2. Detection and Quantification of Pesticides

The determination of 6 pesticides in fish flesh was detected by following multiresidue method [15] using gas chromatograph equipped with electron capture detector having nitrogen (N_2_) at the flow rate of 30–32 mL/minute and with variable temperature arrangements (injector temperature (220°C); oven temperature 150°C maintained for 4 minutes, then raised to 290°C at a rate of 8°C/minute, and then hold for 10 minutes; detector temperature: 300°C). Gas chromatograph was turned on for the detection and quantification of pesticides. First of all 1 *μ*L standard solution of pesticides under this study was injected and their retention times were determined. Calibration curves of all standard pesticides were prepared with the help of computer software Turbochrome and limit of detection was calculated by computer software Super cal-5 (Perkin Elmer, Inc., USA). After running standard solutions of pesticides, 1 *μ*L aliquot of concentrated elute of fish flesh was injected. Residue peak(s) of elute(s) injected was identified on the basis of retention time. The retention time of all test solutions was within ±2% of standard pesticide solutions. Height of residue peak(s) was measured, and residue amount of test solution was determined, by comparison with the height/area obtained from a known amount of appropriate reference/standard solution in the chromatograms.

### 2.2. Statistical Analysis of Data

Data on pesticide residue concentrations in* Catla catla *was analyzed by using two-way classification (factorial experiment). Analysis of Variance and Duncan's Multiple Range tests was performed to analyze differences between the parameters under study [16].

## 3. Results

### 3.1. Dichlorodiphenyltrichloroethane (DDT)

**Figure 2:**
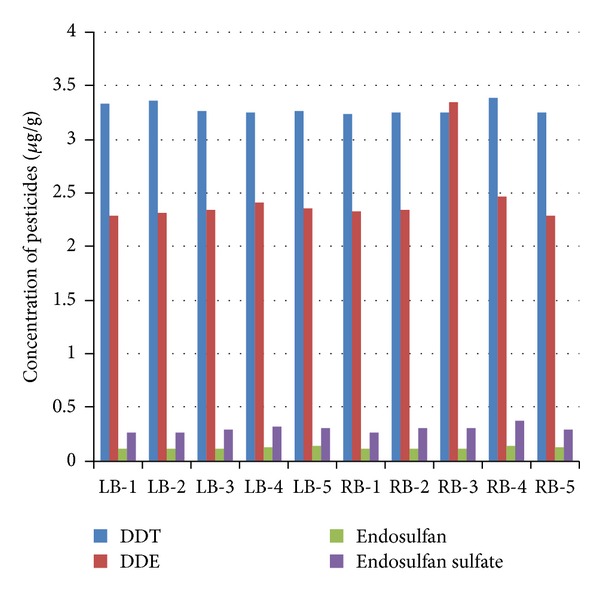
Comparison of different pesticides detected in fish muscle of* Catla catla* from ten different locations of Ravi river.

**Figure 3:**
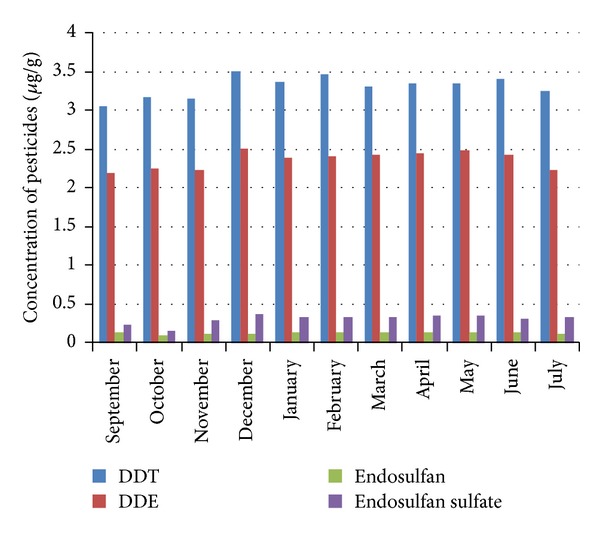
Monthly comparison of pesticides concentration in* Catla catla* collected from Ravi river.

Mean annual DDT concentrations in* Catla catla *fluctuated between a minimum of 3.240 ± 0.0274 *μ*g g^−1^ at Before Hudiara nulla fall (RB) and maximum of 3.389 ± 0.0166 *μ*g g^−1^ at After Degh fall (RB) river sampling sites, respectively. The difference between these two sites, for the concentration of DDT in fish muscle, was statistically significant (*P* < 0.05), while the differences between other river sampling sites, Bakar Mandi nulla fall (LB), Before Hudiara Nulla fall (LB), After Hudiara nulla fall (LB), Balloki Headworks (LB), Shahdara bridge (RB), After Farrukhabad nulla (RB), before Degh fall (RB), and Balloki Headworks (RB), were statistically (*P* > 0.05) nonsignificant (Table 1(c) and Figure 2). The differences in the concentration of DDT in fish muscle between sampling sites Bakar Mandi nulla fall (LB), Balloki Headworks (LB), and Shahdara bridge (RB) were statistically significant (*P* < 0.05). The lowest mean concentration of 3.041 ± 0.010 *μ*g g^−1^ of DDT in* Catla catla *was detected during the month of September 2009, while the highest mean concentration of 3.351 ± 0.004 *μ*g g^−1^ of DDT in* Catla catla *was detected during the month of December 2009, respectively (Table 1(d) and Figure 3). The difference between these months, for the toxicity of DDT, was statistically highly significant (*P* < 0.01).

### 3.2. Dichlorodiphenyldichloroethylene (DDE)

Table 1(c) shows that* Catla catla *collected from Balloki Headworks (RB) river sampling site showed minimum mean annual DDE concentration of 2.290 ± 0.0196 *μ*g g^−1^, while at After Degh fall (RB) river site maximum mean annual DDE concentration of 2.460 ± 0.0340 *μ*g g^−1^ was detected. The difference between these river sampling sites, for the toxicity of DDE, was statistically highly significant (*P* < 0.01). The lowest mean concentration of 2.180 ± 0.015 *μ*g g^−1^ of DDE in* Catla catla *was detected during the month of September 2009, while the highest mean concentration of 2.509 ± 0.014 *μ*g g^−1^ of DDE in* Catla catla *was detected during the month of December 2009, respectively (Table 1(d) and Figure 2). The difference between these months, for the toxicity of DDE, was statistically highly significant (*P* < 0.01).

### 3.3. Endosulfan

Mean annual minimum endosulfan concentration in* Catla catla *was detected as 0.112 ± 0.003 *μ*g g^−1^ at the Shahdara bridge (LB), whereas maximum mean annual concentrations of endosulfan (0.136 ± 0.0031 *μ*g g^−1^) was detected at After Degh fall (RB) river sampling site, respectively (Table 1(c) and Figure 2). The difference between these two river sampling sites, for the toxicity of endosulfan, was statistically highly significant (*P* < 0.01). The lowest mean concentration of 0.099 ± 0.002 *μ*g g^−1^ of endosulfan was detected in* Catla catla *during the month of August 2010, while the highest mean concentration of 0.137 ± 0.003 *μ*g g^−1^ of endosulfan in* Catla catla *was detected during the month of January 2010, respectively (Table 1(d); Figure 3). The difference between these two months, for the toxicity level of endosulfan, was statistically highly significant (*P* < 0.01).

### 3.4. Endosulfan Sulfate

Endosulfan sulfate is a persistent environmental metabolite of endosulfan, an organochlorine insecticide-acaricide. Toxicity is partly dependent on the manner with which the pesticide is administered. Undiluted endosulfan is slowly and incompletely absorbed into the body whereas absorption is more rapid in the presence of alcohols, oils, and emulsifiers.This organochlorine pesticide was not detected in fish muscle of* Catla catla *from all the ten selected sampling locations of the Ravi river.

### 3.5. Carbofuran

The mean annual carbofuran concentrations in* Catla catla *fluctuated between a minimum of 0.260 ± 0.0158 *μ*g g^−1^ at the Shahdara bridge (LB) and maximum of 0.370 ± 0.0191 *μ*g g^−1^ at After Degh fall (RB) river sampling sites, respectively. The difference between these two rivers sampling sites, for the toxicity of carbofuran, was statistically (*P* < 0.01) highly significant (Table 1(c) and Figure 2). The lowest mean concentration of 0.150 ± 0.030 *μ*g g^−1^ in* Catla catla *was detected during the month of October 2009, while the highest mean concentration of 0.360 ± 0.010 *μ*g g^−1^ of carbofuran in* Catla catla *was detected during the month of December 2009, respectively (Table 1(d) and Figure 3). The difference between these two months, for the toxicity level of endosulfan, was statistically highly significant (*P* < 0.01).

### 3.6. Cartap

Cartap hydrochloride is a nereistoxin analog and is a commonly used low-toxicity insecticide. It is commonly used as a hydrochloride (C_7_H_15_N_3_O_2_S_3_HCl). Cartap is essentially a contact and stomach poison. It is used for the control of chewing and sucking pests and results in insect paralysis. It has been categorized as a high-effectiveness, low-toxicity, and low-residue pesticide used in rice and sugarcane fields. Cartap was not detected in fish muscle of* Catla catla* from all the ten selected sampling locations of the Ravi river.

The levels of pesticides were below the tolerance limits suggested in national and international standards. Pesticide concentrations in the water of river sites ranged from 0.034 to 0.045 *μ*g/L for DDT, 0.033 to 0.046 *μ*g/L for DDE, 0.108 to 0.123 *μ*g/L for endosulfan, and 0.028 to 0.040 *μ*g/L for carbofuran. In the tributaries, pesticide concentrations ranged from 0.0468 to 0.0685 *μ*g/L for DDT, 0.0390 to 0.0637 *μ*g/L for DDE, 0.111 to 0.147 *μ*g/L for endosulfan, and 0.0396 to 0.0631 *μ*g/L for carbofuran (Figures 2 and 3). The results show that the pesticide concentrations in river water decreased in the order: endosulfan > DDE > DDT > carbofuran and pesticide concentrations in tributary waters decreased in the order: endosulfan > DDT > DDE > carbofuran. After Degh fall and After Hudiara nulla fall, river sampling sites were severely contaminated while, among the tributaries, Degh fall and Hudiara drain were severely contaminated with DDT, DDE, endosulfan, and carbofuran. A constant monitoring programs should be initiated to reform the present situation.

## 4. Discussion

Toxic and environmentally persistent agrochemicals have been used extensively all over the world for very long and are being used these days [17, 18]. Indiscriminate use of such chemicals has resulted in extreme damage to humans and the environment [5]. Although developed world and some developing countries have banned the use of these persistent pollutants for the last 25 years due to their biomagnifications in the food chain, and findings of a number of research studies such as conducted by [19, 20] and surveys [21, 22] witness the presence of these banned pesticides in water, sediments/soil, flora and fauna of lakes, streams, rivers, and other water reservoirs especially in developing countries. Pakistan has also imposed ban on the use of organochlorine pesticides, but the research studies conducted in the last decade reported their continuous use [12, 14]. The presence of these pesticides despite the ban on their use is due to their stability, long half lives, lack of adequate regulation and management on the production, and trade and usage of these agrochemicals [11]. Still continuous use of organochlorine pesticides in the developing countries might be due to their lower cost and high efficacy as compared to other alternative pesticides [5] and the present study also showed the presence of these OCPs in fish.

In the present study fish samples of* Catla catla *collected from all the ten collection sites from the Ravi river were found contaminated with the organochlorine and nitrogen containing pesticides (DDT, DDE, endosulfan, and carbofuran). Out of ten sampling sites on Ravi river, After Degh fall river sampling site (RB), was found with the highest pesticide contamination level in fish samples followed by After Hudiara drain fall river sampling site (LB).* Catla catla *collected from After Degh fall river sampling site (RB) showed the highest contamination level of 3.389 ± 0.0166 *μ*g g^−1^ of DDT, 2.460 ± 0.0340 *μ*g g^−1^ of DDE, 0.136 ± 0.0031 *μ*g g^−1^ of endosulfan, and 0.370 ± 0.0191 *μ*g g^−1^ of carbofuran, respectively. The maximum level of pesticide contamination at after Degh fall river sampling site (RB) is due to the entry of Degh fall nulla into Ravi River. After Degh fall river sampling site (RB) was found severely contaminated by pesticides probably this tributary is bringing a lot of untreated waste from various sources. This is supported by a study on the chemical contaminants in water and sediments of Degh nulla by [23], who reported that organochlorine pesticides, especially DDT and DDE, were found in higher limits than maximum residue limits (MRLs). Although contamination levels of endosulfan and carbofuran were not exceeding the MRL as set by the Codex Alimentarious Commission of [22], but DDT and DDE concentrations were more than the MRLs for food standards set by the Codex Alimentarious Commission of [22]. The findings of [22] are in line with the present study as they reported that a number of organochlorine pesticide residues including p,p′-DDE, o,p′-DDE, p,p′-DDT, o,p′-DDT, endosulfan, endosulfan sulfate, Dieldren, Heptachlor and Lindane were detected by liquid-liquid extraction was found higher than allowed limits of EPA, followed by gas chromatograph-mass spectrophotometer technique in samples taken seasonally from Selangor river in Malaysia and the present study is also in agreement with the findings of [14]. The levels of pesticide residues above than MRLs in fish are due to the lack of updated food regulations in Pakistan. Unfortunately, no attention has been paid by the concerned authorities to implement the food laws in the country. Futhermore, pure food laws of Pakistan are approximately 50 years old and have no practical applications for the pesticide residue limits in food items [24]. No prescribed maximum residue limits for various food commodities are till to date set by the concerned authorities to ensure the safely supply of food items including the fish for human consumption in the country.

In the present study, maximum contamination levels of pesticides in fish samples were detected during the months of low water supply (dry season) of the year, especially December 2009 and January–May 2010, when less water was flowing in Ravi River. It is due to the fact that, during these low water supply months, pesticides get concentrated in water and sediments of river bodies and chances for the contamination of fish and other flora and fauna increase and maximum level of pesticide was detected in muscle of* Catla catla*. The above findings are in agreement with the findings of [18]. Reference [25] also reported high OCPs residue level during dry season. Months of summer, especially of rainy season such as September 2009, July 2010, and August 2010, were found with minimum contamination levels of pesticides in fish samples of Ravi River because during these months, water supply was increased in the river due to heavy rains. Higher pesticide levels were recorded in the dry season in fish muscle samples. This could be attributed to different living habits of the fish between the seasons, breaking down of the DDT, DDE, endosulfan, and endosulfan sulfate in the environment, and to its absorption through the skin. According to [26], accumulation of organic contaminants in the tissue of aquatic organisms depends on the physicochemical properties of the contaminant, its distribution in the aquatic system, and the feeding behaviour and metabolism of the aquatic organism. The more amount of water in rivers, nullas, and drains dilutes the pesticides up to maximum extent and chances for the contamination of fish, sediments, and water decrease and minimum level of pesticides was detected. Moreover, during the rainy season water flows with great speed which does not permit pesticides to get deposited in sediments of water bodies, which further reduce the chances for contamination of fish, sediments, and water.

Organochlorine water contamination pathways in the Ravi river are likely to be nonpoint sources, including runoff, atmospheric deposition, and leaching due to agricultural applications and vector control practices. Sediments can act as a sink for persistent contaminants, whose resuspension at the sediment-water interface, especially in storm events and during river mixing, increased pesticide bioavailability and accumulation in the food chain in particular DDT and DDE. Pesticide pollution is, therefore, likely to pose a threat to both aquatic organisms and humans. Generally, it is believed that contaminants taken in by aquatic organisms are from water, rather than from their food, and may vary with seasonal variation in contaminant availability within the water column [5]. There was a general increasing trend of the concentrations of the pollutants as the river progresses Hudiara drain to Head Balloki. Areas near the large scale cash crop farms (cotton crop, rose flowers, sugar cane, and rice) were the most polluted. Organochlorine water contamination pathways in the Ravi river are likely to be nonpoint sources and which are gradually increasing day by day.

## 5. Conclusion

It was concluded that pesticide toxicity level of Ravi River is due to the pesticide toxicity levels of its tributaries. These tributaries carry a huge burden of concentrated industrial, domestic, and agricultural waste substances and pour them as such into the main river water. Unfortunately, before the fall of these tributaries into the Ravi river, no treatment for the removal or breakdown of pollutants is done. The stretch of Ravi River from Shahdara to Head Balloki has been found polluted and rendering the water unfit for aquatic life. Awareness should be developed among the general public through the print and electronic media so that they may realize the harmful effects of pesticides and stop/reduce their use. Currently, a constant monitoring program needs to be initiated to reform the present situation.

## Figures and Tables

**Table 1a:** (a)

S.O.V	D.F1	*F*-Values			
		DDT	DDE	Endosulfan	Carbofuran
Months	11	650.88**	395.44**	185.54**	30.81**
Sampling Site	9	14.22**	61.12**	115.33**	8.24**
Months × Sites	9	2028**	4.90**	22.71**	0.28^NS^

**Highly Significant (*P* < 0.01); NS = nonsignificant (*P* > 0.05).

**Table 1b:** (b)

S.E	DD	DDE	Endosulfan	Carbofuran
Months	0.0049	0.0071	0.0009	0.0129
Sampling Sites	0.005	0.0062	0.0008	0.012
Months × Sites	0.016	0.0211	0.0031	0.0390

**Table 1c:** (c) Sampling sites wise comparison of means (*μ*g/g ± SE)

Sampling station	DDT	DDE	Endosulfan	Carbofuran
LB-1 = Shahdara bridge	3.331 ± 0.0246^C^	2.292 ± 0.0249^E^	0.112 ± 0.0031^F^	0.260 ± 0.0158^C^
LB-2 = Bakar Mandi nulla fall	3.359 ± 0.0239^BC^	2.317 ± 0.0234^DE^	0.116 ± 0.0027^EF^	0.265 ± 0.0134^BC^
LB-3 = Before Hudiara nulla fall	3.270 ± 0.0235^BC^	2.340 ± 0.0270^CD^	0.119 ± 0.0021^CD^	0.297 ± 0.0160^BC^
LB-4 = After Hudiara nulla fall	3.250 ± 0.0235^B^	2.411 ± 0.0296^B^	0.131 ± 0.0057^B^	0.314 ± 0.0161^B^
LB-5 = Balloki Headworks	3.270 ± 0.0234^BC^	2.351 ± 0.0305^BC^	0.135 ± 0.0051^B^	0.304 ± 0.0171^BC^
RB-1 = Shahdara bridge	3.240 ± 0.0274^BC^	2.322 ± 0.0274^DE^	0.112 ± 0.0031^F^	0.270 ± 0.0150^BC^
RB-2 = After Farrukhabad nulla	3.250 ± 0.0229^BC^	2.346 ± 0.0268^BC^	0.116 ± 0.0032^DE^	0.299 ± 0.0150^BC^
RB-3 = Before Degh fall	3.247 ± 0.0220^BC^	2.341 ± 0.0263^BC^	0.118 ± 0.0020^D^	0.312 ± 0.0170^AB^
RB-4 = After Degh fall	3.389 ± 0.0166^A^	2.460 ± 0.0340^A^	0.136 ± 0.0031^A^	0.370 ± 0.0191^A^
RB-5 = Balloki Headworks	3.255 ± 0.0214^B^	2.290 ± 0.0196^E^	0.121 ± 0.0028^C^	0.292 ± 0.0160^BC^

**Table 1d:** (d) Month wise comparison of means (*μ*g/g ± SE)

Months	DDT	DDE	Endosulfan	Carbofuran
September 2009	3.041 ± 0.010^G^	2.180 ± 0.015^H^	0.127 ± 0.004^C^	0.230 ± 0.009^D^
October 2009	3.170 ± 0.008^D^	2.255 ± 0.011^F^	0.095 ± 0.002^F^	0.150 ± 0.030^E^
November 2009	3.144 ± 0.005^E^	2.234 ± 0.004^F^	0.116 ± 0.004^E^	0.290 ± 0.006^C^
December 2009	3.351 ± 0.004^A^	2.509 ± 0.014^A^	0.120 ± 0.003^D^	0.360 ± 0.010^A^
January 2010	3.355 ± 0.002^A^	2.392 ± 0.027^E^	0.137 ± 0.003^A^	0.321 ± 0.015^AB^
February 2010	3.347 ± 0.003^A^	2.405 ± 0.012^DE^	0.136 ± 0.011^AB^	0.333 ± 0.013^AB^
March 2010	3.299 ± 0.012^B^	2.419 ± 0.017^D^	0.129 ± 0.004^C^	0.336 ± 0.009^AB^
April 2010	3.344 ± 0.004^A^	2.440 ± 0.016^C^	0.134 ± 0.004^B^	0.352 ± 0.006^A^
May 2010	3.340 ± 0.003^A^	2.480 ± 0.013^B^	0.134 ± 0.006^B^	0.350 ± 0.005^A^
June 2010	3.341 ± 0.005^A^	2.432 ± 0.018^C^	0.130 ± 0.002^B^	0.308 ± 0.011^BC^
July 2010	3.238 ± 0.003^C^	2.220 ± 0.003^F^	0.116 ± 0.002^D^	0.339 ± 0.009^AB^
August 2010	3.097 ± 0.013^F^	2. 2.186 ± 0.007^G^	0.099 ± 0.002^G^	0.234 ± 0.008^D^

Means sharing similar letters in a single column are statistically nonsignificant (*P* > 0.05).
